# The Shade Avoidance Syndrome in Arabidopsis: The Antagonistic Role of Phytochrome A and B Differentiates Vegetation Proximity and Canopy Shade

**DOI:** 10.1371/journal.pone.0109275

**Published:** 2014-10-21

**Authors:** Jaime F. Martínez-García, Marçal Gallemí, María José Molina-Contreras, Briardo Llorente, Maycon R. R. Bevilaqua, Peter H. Quail

**Affiliations:** 1 Institució Catalana de Recerca i Estudis Avançats, Barcelona, Spain; 2 Centre for Research in Agricultural Genomics (CRAG), Consortium CSIC-IRTA-UAB-UB, Barcelona, Spain; 3 CAPES foundation, Ministry of Education of Brazil, Brasilia - DF, Brazil; 4 Department of Plant and Microbial Biology, University of California, Berkeley, California, United States of America; 5 US Department of Agriculture/Agriculture Research Service, Plant Gene Expression Center, Albany, California, United States of America; University of Texas at Austin, United States of America

## Abstract

Light limitation caused by dense vegetation is one of the greatest threats to plant survival in natural environments. Plants detect such neighboring vegetation as a reduction in the red to far-red ratio (R:FR) of the incoming light. The low R:FR signal, perceived by phytochromes, initiates a set of responses collectively known as the shade avoidance syndrome, intended to reduce the degree of current or future shade from neighbors by overtopping such competitors or inducing flowering to ensure seed production. At the seedling stage these responses include increased hypocotyl elongation. We have systematically analyzed the Arabidopsis seedling response and the contribution of phyA and phyB to perception of decreased R:FR, at three different levels of photosynthetically active radiation. Our results show that the shade avoidance syndrome, induced by phyB deactivation, is gradually antagonized by phyA, operating through the so-called FR-High Irradiance Response, in response to high FR levels in a range that simulates plant canopy shade. The data indicate that the R:FR signal distinguishes between the presence of proximal, but non-shading, neighbors and direct foliar shade, via a intrafamily photosensory attenuation mechanism that acts to suppress excessive reversion toward skotomorphogenic development under prolonged direct vegetation shade.

## Introduction

The shade avoidance syndrome (SAS) refers to a set of plant responses aimed at adapting plant growth and development to high plant density environments, like those found in forests, prairies or orchard communities. Two related but different situations can occur in these environments: plant proximity (without direct vegetative shading) and direct plant canopy shade [1]–[3]. Because vegetation preferentially reflects far-red (FR) light compared to other wavelengths, plant proximity generates a reduction in the red (R, about 600–700 nm) to far-red (FR, between 700–800 nm) ratio (R:FR) in the light impinging on neighbors. By contrast, under a plant canopy, light from the visible region (called photosynthetically active radiation or PAR, between 400–700 nm) is strongly absorbed by the chlorophyll and carotenoid photosynthetic pigments whereas FR, which is poorly absorbed by the leaves, is transmitted through (or reflected from) vegetation. As a consequence, under direct plant canopy shade both the amount of PAR (light quantity) and R:FR (light quality) are greatly reduced, in the latter case mostly by the selective depletion of R light caused by the filtering of sunlight through the leaves [1], [2], [4]–[6].

This low R:FR signal is perceived by the phytochrome (phy) photoreceptors. Phys detect the R and FR part of the spectrum and have a major role in controlling several adaptive responses such as seed germination, stem elongation, leaf expansion and flowering time. In Arabidopsis (*Arabidopsis thaliana*), a small gene family of five members encodes the phys (*PHYA*-*PHYE*) [7]. Although phyB is the major phy controlling the SAS, genetic and physiological analyses have shown that other phys act redundantly with phyB in the control of some aspects of SAS-driven development, such as flowering time (phyD, phyE), petiole elongation (phyD, phyE) and internode elongation between rosette leaves (phyE) [2], [4]. The photolabile phyA has the unique capacity to function as a FR-light sensor through a mechanism termed the FR-High Irradiance Response (HIR) [2], [8], [9]. In contrast to the other phys, phyA has an antagonistic negative role in the SAS hypocotyl response, although varying degrees of regulation have been reported: *phyA* mutant seedlings growing under low R:FR light showed from moderately [10] to extremely long hypocotyls [11]. An antagonistic activity between phyA and phyB has also been shown in seedlings exposed to varying ratios of monochromatic R and FR [12], [13]. However, it has been argued that the adaptive significance of this phyA antagonism is limited and may instead be an inevitable consequence of the intrinsic properties of phyA selected for their role in seedling deetiolation [2].

Phys exist in two photoconvertible forms, an inactive R-absorbing Pr form and an active FR-absorbing Pfr form. In light-grown plants, the steady-state ratio of Pr and Pfr conformers depends on the R:FR ratio. Under high R:FR the photoequilibrium is displaced towards the active Pfr form and the SAS is suppressed. Under low R:FR the photoequilibrium is displaced towards the inactive form and SAS is induced. This induction is regulated at least partly by the interaction of active phys with various PHYTOCHROME INTERACTING FACTORs (PIFs) [14]–[16], which results in rapid changes in the expression of dozens of *PHYTOCHROME RAPIDLY REGULATED* (*PAR*) genes, postulated to be instrumental in implementing the SAS responses [17]–[21]. Because many of these *PAR* genes encode transcriptional regulators, it is assumed that shade responses are a consequence of the phy regulation of a complex transcriptional network, as postulated for seedling de-etiolation [22], [23], that seems to be organized in functional modules [24]. Genetic analyses demonstrated positive and negative roles in SAS regulation for several *PAR* genes encoding transcriptional regulators, including members of the homeodomain leucine zipper class II (*ATHB2*, *ATHB4*, *HAT1*, *HAT2* and *HAT3*), basic-helix-loop-helix (*BEE1*, *BEE2*, *BIM1*, *BIM2*, *HFR1*, *PAR1*, *PAR2* and *PIL1*) and B-BOX CONTAINING (BBX) families of proteins [17]–[19], [24]–[30]. Most of these studies were done analyzing hypocotyl elongation. Therefore, low R:FR perception rapidly changes the balance of positive and negative factors, resulting in the appropriate SAS responses, i.e., eventually causing hypocotyls to elongate. Evidence for the involvement of several of these factors in controlling auxin levels and sensitivity in mediating this elongation response has been reported [16], [31], [32].

The light treatments used to induce the SAS vary among laboratories, resulting in differences in the extent of the responses (usually hypocotyl length) reported for the same genotype. For instance, a review of several papers in the field reported that Arabidopsis Col-0 hypocotyls elongate in response to low R:FR (under laboratory conditions usually provided by white light supplemented with FR light, W+FR) from a minimum of about 2.5 mm to a maximum of ca. 9 mm [15], [20], [26], [28], [33]–[35]. In addition to media composition, variations in the timing and nature of the W+FR treatment might also explain some of the observed differences. For instance, the reported effect of the negative SAS regulator HFR1 on the shade-induced hypocotyl elongation ranged from mild [25] to very strong [27]. We have noted previously that the very strong phenotype of *hfr1* mutant seedlings was observed under shade conditions that reduced both R:FR and PAR (400–700 nm), whereas the mild phenotype occurred under shade conditions where only the R:FR ratio was reduced without significantly affecting the PAR [25]. Indeed, although the SAS is generally considered to be mainly induced by light of reduced R:FR, other light parameters, such as low-intensity light of the whole PAR spectrum and low blue light (which is part of the PAR spectrum), are also known to contribute to these responses [6], [36]–[38]. Together, these observations highlight the fact that different shade conditions (such as variable PAR and/or R:FR) employed by different labs might account for some of the observed variability in the SAS response.

In this paper we have investigated the effect of both the level of PAR and supplemental FR (which results in different R:FR ratios without altering PAR) in the incoming light on hypocotyl elongation. To address the contribution of the two major phys in this response, we have systematically analyzed wild-type, *phyA* and *phyB* mutant seedlings. We observe that, independently of the PAR level employed, the R:FR ratio strongly and differentially affects elongation of wild-type, *phyA* and *phyB* hypocotyls. Our results indicate that quantitative variation in the R:FR ratio provides a dual signal with a likely different meaning in nature: when the R:FR is moderately lowered, it mimics plant proximity without direct shading, whereas when it is very low, it mimics direct plant canopy shade. In addition, the effects of these two environmental conditions can be distinguished genetically, with phyA and phyB having different roles in transducing the signals, as shown previously for seedling de-etiolation.

## Materials and Methods

### Plant material and growth conditions

Arabidopsis (*Arabidopsis thaliana*) plants for seed production were grown in the greenhouse as described [39]. The *phyA-501* (SALK_014575) [40] and *phyB-9* [41] mutant lines are in Col-0 ecotype. The *phyA* and *phyB* mutant lines in L*er* have been described previously [42]. Homozygous *phyA-501* plants were genotyped as indicated in Figure S1 by using specific oligos: MSO31 (5′–TAG-AGC-ACC-GCA-CAG-CTG-CC-3′), MSO32 (5′– GAA-GCT-ATC-TCC-TGC-AGG-TGG– 3′) and LBb1 (5′-GCG-TGG-ACC-GCT-TGC-TGC-AAC-T-3′).

**Figure 1:**
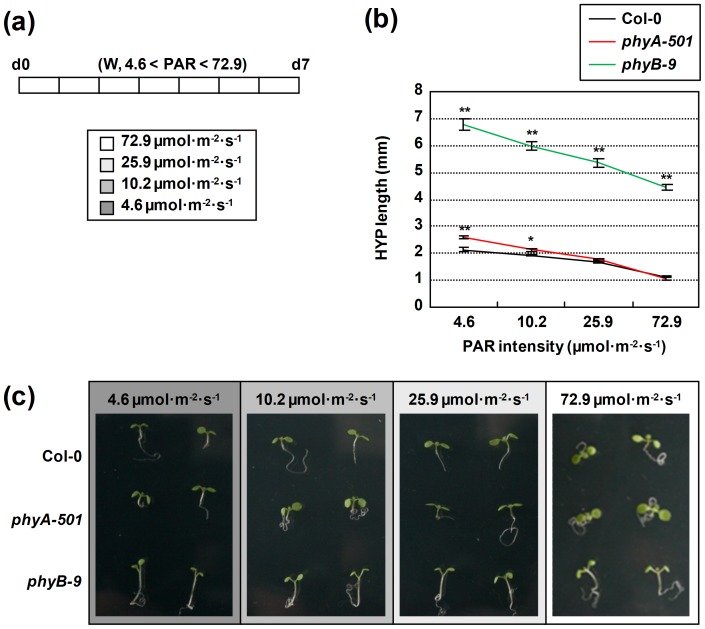
Effect of increasing intensities of white light on hypocotyl elongation of wild-type, *phyA-501* and *phyB-9* seedlings. (**a**) Seedlings of the indicated genotypes were grown from the day of germination until day 7 under W of increasing intensities (photosynthetic active radiation, PAR, between 4.6 and 72.9 µmol·m^−2^·s^−1^; R:FR>2.0). (**b**) Hypocotyl length of seedlings grown as indicated in **a**. Values are means ± SE of at least 25 hypocotyls for each light treatment. Asterisks indicate significant differences (*P<0.05, **P<0.01) relative to the control grown under the same light intensity. (**c**) Representative seedlings, grown as indicated in **a**, are shown for the three genotypes analyzed.

**Figure 2:**
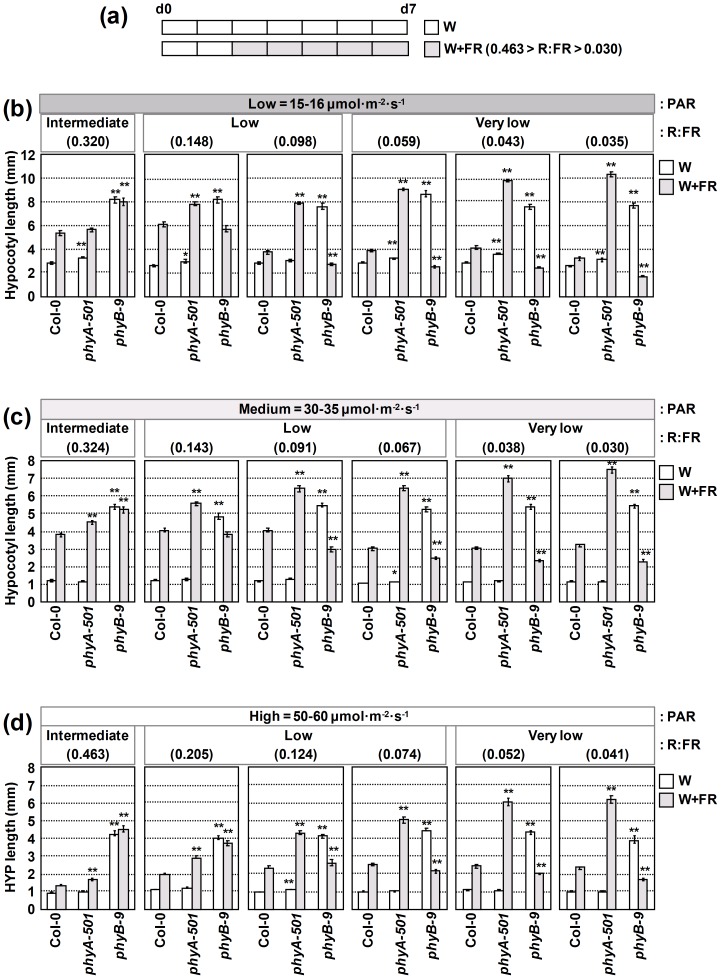
Effect of different R:FR on hypocotyl elongation of wild-type, *phyA-501* and *phyB-9* seedlings under low, medium or high PAR. (**a**) Seedlings were germinated and grown for 2 days under W light and then either kept in W or transferred to W supplemented with increasing amounts of FR for 5 more days. Hypocotyl length of seedlings grown as indicated in **a** under (**b**) low, (**c**) medium and high (**d**) PAR. The amount of PAR is given at the top of each section. The type of R:FR applied (nomenclature provided in Table S1) in the given W+FR treatments is indicated at the top, of the graphs; the R:FR value of each experiment is indicated at the top of each graph. In **b**, **c** and **d**, values are means ± SE of at least 25 hypocotyls for each light treatment. Asterisks indicate significant differences (*P<0.05, **P<0.01) relative to the control (Col-0) grown under the same light conditions.

**Figure 3:**
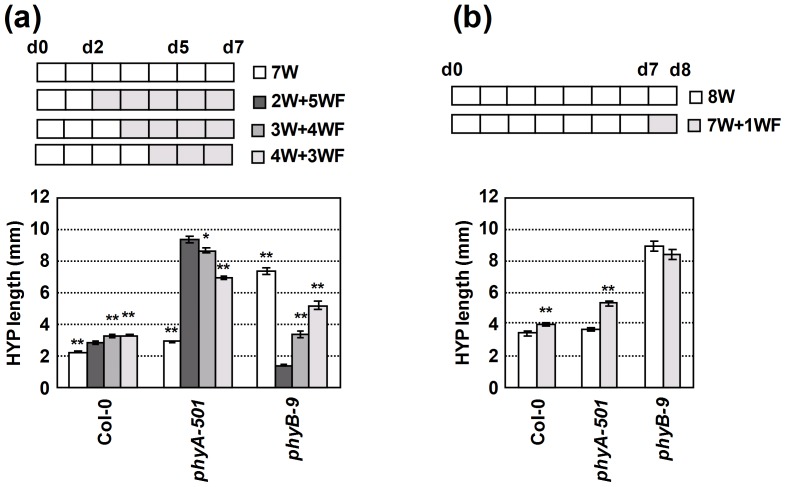
Effect of time of W+FR treatment on hypocotyl elongation of wild-type, *phyA-501* and *phyB-9* seedlings under low PAR. (**a**) Hypocotyl length of seedlings germinated and grown for 2, 3, 4 or 7 days under W light and then transferred to W+FR for 5, 4, 3 or 0 more days, respectively. (**b**) Hypocotyl length of seedlings germinated and grown for 7 days under W light and then either kept in W or transferred to W+FR for 1 more day. PAR was of 15–16 µmol·m^−2^·s^−1^ and R:FR of 0.059. Values are means ± SE of at least 25 hypocotyls for each light treatment. Asterisks indicate significant differences (*P<0.05, **P<0.01) relative to the same genotype (Col-0) grown for 2 days under W and 5 days under W+FR.

**Figure 4:**
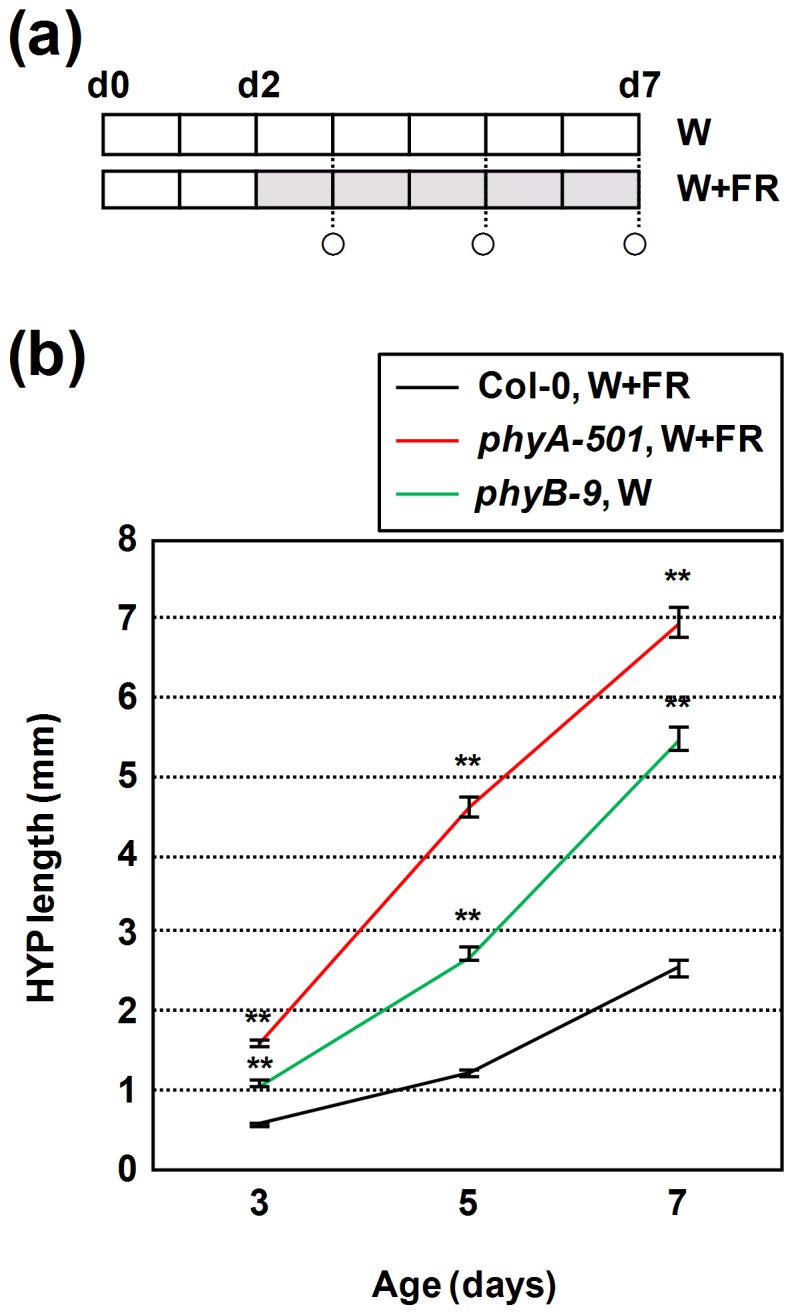
Effect of *phyA* and *phyB* mutations on the temporal evolution of the hypocotyl length. (**a**) Seeds were germinated and grown for 2 days under W (PAR was of 20–25 µmol·m^−2^·s^−1^) and then either kept under W (*phyB* seedlings) or transferred to W+FR (R:FR = 0.038) for 5 more days (Col-0 and *phyA* seedlings). Circles indicate the days on which hypocotyls were measured. (**b**) Hypocotyl length of seedlings grown as indicated in **a**. Values are means ± SE of at least 25 hypocotyls for each light treatment. Asterisks indicate significant differences (*P<0.05, **P<0.01) relative to the wild type seedlings grown under the corresponding light conditions.

**Figure 5:**
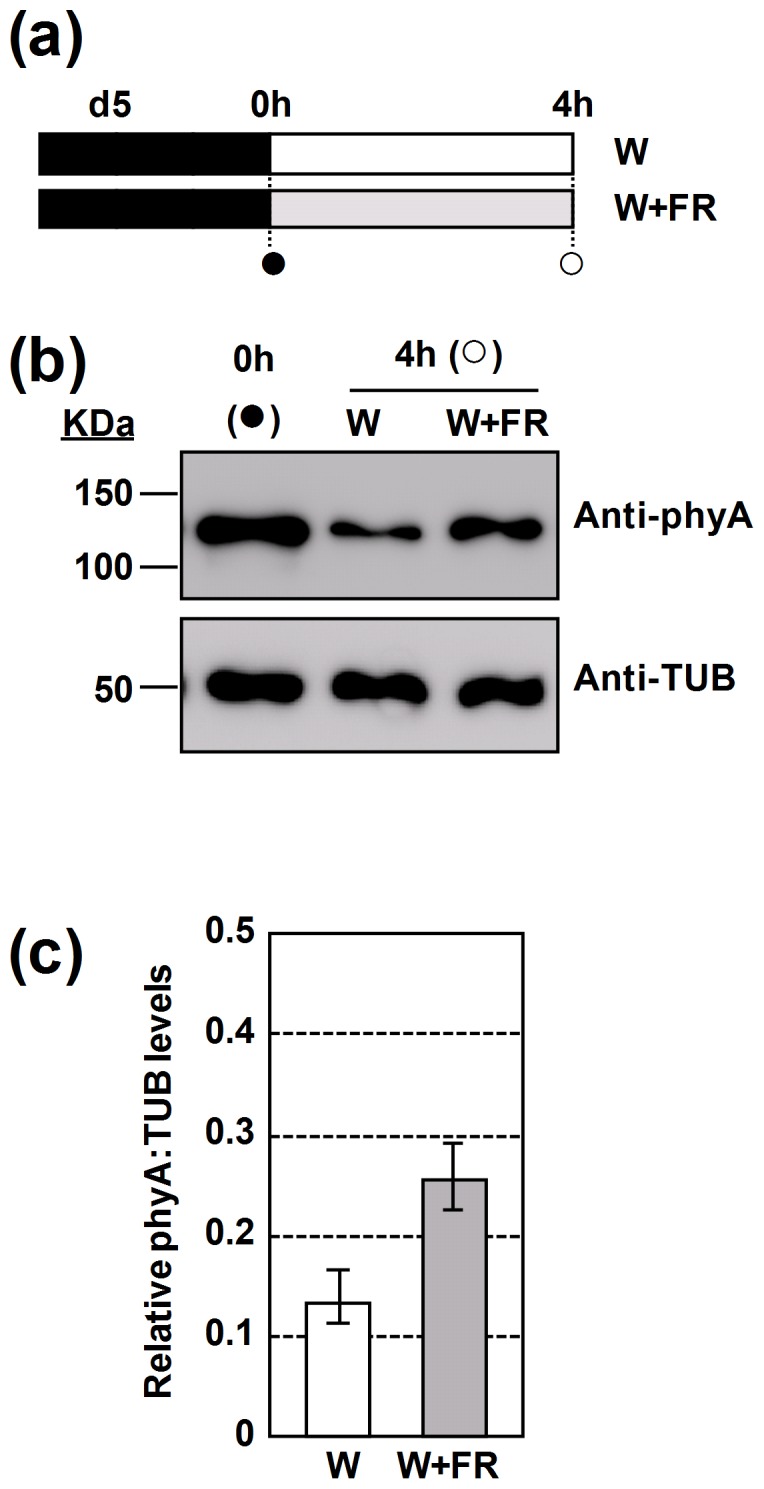
Phytochrome A is stabilized by white light of very low R:FR. (**a**) phyA levels were assayed in extracts from Col-0 seedlings grown in darkness for 5 days and then either transferred to W or W+FR for 4 hours. Circles indicate the harvest time of the plant material. (**b**) Representative steady-state levels of phyA (upper panel) and tubulin (TUB, lower panel) in extracts from seedlings grown as indicated in **a**,. Bands were detected by immunoblot using the phyA-specific mAb 073D or a TUB-specific mAb. TUB was used as a loading control. (**c**) Relative levels of phyA normalized to TUB in seedlings differentially grown for 4 h under W or W+FR, as indicated in **a**; n = 3 independent biological replicas; the P-value between the W and W+FR treated samples was 0.053.

All the experiments were performed with seeds surface-sterilized and sown on Petri dishes with solid growth medium without sucrose (GM–; 0.215% (w/v) MS salts plus vitamins, 0.025% (w/v) MES pH 5.8) [17]. After stratification (3–6 days), plates were incubated in growth chambers at 22°C under continuous W that was provided by 2–4 cool-white horizontal fluorescent tubes (Figures 1–3), unless otherwise stated. These tubes delivered different amounts of photosynthetically active radiation (PAR), and a R:FR of about 2.5. In Figures 4 and 5, plates were incubated in growth chambers at 22°C under continuous W provided by 4 cool-white vertical fluorescent tubes (PAR of 20–25 µmol·m^−2^·s^−1^, R:FR of about 2.5). Simulated shade (W+FR) was generated by enriching W with supplementary FR provided by LED lamps (www.quantumdev.com; or www.philips.com/horti). Unless otherwise stated, fluence rates and PAR were measured with a LI-1800 spectroradiometer (Li-Cor Inc., www.licor.com); to calculate the R:FR, windows of 30 nm around the R (640–670 nm) and FR (720–750 nm) peaks were employed. For Figure 4, 5, and S4, fluence rates were measured with a Spectrosense2 meter associated with a 4-channel sensor (Skye Instruments Ltd., www.skyeinstruments.com), which measures PAR (400–700 nm) and 10 nm windows in the R (664–674 nm) and FR (725–735 nm) regions.

### Hypocotyl length measurements

The National Institutes of Health ImageJ software (Bethesda, MD, USA; http://rsb.info.nih.gov/) was used on digital images to measure the length of hypocotyl seedlings (after laying out seedlings flat on agar plates) as indicated elsewhere [26]. At least 25 seedlings were used for each treatment. Experiments were repeated 3–5 times and a representative one is shown. Statistical analyses of the data (t-test) were performed using GraphPad Prism version 4.00 for Windows (www.graphpad.com/) or Excel.

### Protein extraction and Western blot analyses

Extracts were prepared following the direct extract protocol indicated elsewhere [43] with the modifications described below. Extracts shown in Figure S1 were prepared from Arabidopsis Col-0 and *phyA-501* seedlings germinated and grown in the dark for 5 days. Fifteen seedlings from each genotype were harvested and placed in 1.5 mL Eppendorf tubes containing 150 µL of (1×) Laemmli buffer. Extracts shown in other Figures were prepared from Arabidopsis Col-0 seedlings germinated and grown in the dark for 5 days and then exposed to 4 and 8 h of W or W+FR. On the day of harvest, 20 seedlings from each treatment were harvested and placed in 1.5 mL Eppendorf tubes containing 300 µL of Laemmli buffer supplemented with protease inhibitors (10 µg/mL Aprotinin, 1 µg/mL E-64, 10 µg/mL Leupeptin, 1 µg/mL Pepstatin A, 10 µM PMSF). Plant material was ground using disposable grinders in the Eppendorf tube at room temperature until the mixture was homogeneous (usually less than 15 s). Once all the samples were prepared, tubes were placed in boiling water for 3 minutes. Tubes were centrifuged in a microfuge at maximum speed (13000 *g*, 10 min) immediately before loading. Fifteen µL of each extract, equivalent to about 1.5 (Figure S1) or 1 seedling (Figures 5, S4), were loaded per lane in an SDS - 8% PAGE. Immunoblot analyses of phyA and TUB were performed as indicated [43] with some minor changes. Mouse monoclonal antibody (mAb) 0.73D, that recognizes phyA from both monocots and dicots [43], were used at 1∶5000 dilutions. Membranes were stripped and rehybridized with a commercial mouse mAb against α-tubulin (www.sigmaaldrich.com) at a 1∶10000 dilution. Anti-mouse horseradish peroxidase-conjugated antibody (www.promega.com) was used as a secondary antibody. ECL or ECL-plus chemiluminescence kits (www3.gehealthcare.com) were used for detection.

### Accession numbers

Sequence data from this paper can be found in the Arabidopsis Genome Initiative or GenBank/EMBL databases under the following accession numbers: *PHYA* (At1g09570) and *PHYB* (At2g18790).

## Results

### The intensity of continuous white light affects seedling morphology

For this study, we have systematically analyzed the hypocotyl response of wild-type (Col-0), *phyA* and *phyB* mutant seedlings to different light conditions. As a phyB-deficient line, we have employed *phyB-9*, a well characterized line [41]. As a phyA-deficient line we have employed a SALK line (SALK_014575) that contains a T-DNA insertion in the middle of the first intron of the *PHYA* gene, about 1260 bp downstream of the ATG start codon (Figure S1a) [40]. In etiolated mutant seedlings, no levels of phyA were detected, whereas tubulin levels were similar to those of wild type (Figure S1b). This line was blind to continuous monochromatic FR, whereas it was as responsive to monochromatic R light as the wild type (Figure S1c). Together, these results confirm that this T-DNA insertion line is a null mutant for *PHYA*. We named this new allele as *phyA-501*.

It is already known that light intensity affects seedling development, particularly hypocotyl length [20]. To get different light intensities, we employed neutral filters that reduced the intensity of white (W) light provided by 4 fluorescent tubes. As a result, PAR ranged from a minimum of 4.6 µmol·m^−2^·s^−1^ to a maximum of 72.9 µmol·m^−2^·s^−1^ (with no filters) (Figure 1a). As expected, hypocotyl elongation of wild-type, *phyA* and *phyB* seedlings was decreased when PAR amount was increased. Hypocotyls of *phyB* seedlings were always longer than those of wild-type and *phyA* seedlings under all different light intensities, as previously reported [41]. By contrast, *phyA* hypocotyls were generally longer than Col-0 at lower light intensities, whereas at high light intensities the differences in length were reduced or abolished (Figure 1b). Additional differences were evident in other morphological traits of the seedlings of the three genotypes analyzed: low light intensities reduced cotyledon expansion and delayed primary leaf development (Figure 1c). No higher PAR conditions were applied because under the highest light intensity employed here (i.e., about 73 µmol·m^−2^·s^−1^ from the beginning of germination) some seedlings showed signs of stress, such as a purple color and small size (data not shown).

### Light of different R:FR differentially affects the hypocotyl elongation of wild-type, phyA and phyB seedlings

Next, we addressed the influence of light with varying reductions in R:FR on hypocotyl length. To manipulate the R:FR, W light of a fixed PAR was enriched with increasing fluence rates of FR light. We started our experiments with a relatively low level of W light, 15–16 µmol·m^−2^·s^−1^ in the PAR region. For simplicity, from now on we will refer to this intensity of W light as “low PAR”. When supplementing with FR, the applied R:FR ranged from 0.320 to 0.035 depending on the amount of FR provided (Figure 2a). Whereas W light provided a high R:FR (>1.5), W+FR provided light with moderate (intermediate R:FR of 0.5-0.3), substantial (low R:FR of 0.29-0.06) and very large (very low R:FR of 0.05-0.03) decrease in R:FR (see Table S1 for nomenclature used here). At the highest R:FR applied (0.320), W+FR light strongly induced hypocotyl elongation of Col-0 (about 5 mm) compared to the W-grown seedlings (about 2.5 mm). Further reductions in the R:FR were first slightly more effective (about 6 mm, R:FR of 0.148), and then rapidly less effective, in promoting hypocotyl elongation (about 4 mm for R:FR between 0.098-0.043), until shade-induced hypocotyl elongation was almost abolished (about 3.3 mm for the lowest R:FR tested of 0.035) (Figures 2b, S2a). These results indicate that quantitative variation in the R:FR provides a dual signal: when the R:FR is moderately or substantially lowered (R:FR of 0.320-0.148), it strongly induces hypocotyl elongation of Col-0, whereas when it is strongly reduced (R:FR<0.043; we define this range of R:FR as “very low R:FR”) it is less effective in promoting the elongation of Col-0 hypocotyl elongation. At the highest R:FR tested (0.320), W+FR light induced the hypocotyl length of *phyA* to an extent similar to that observed for Col-0. But in striking contrast to the behavior of the Col-0 seedlings, the progressive reduction of the R:FR resulted in a gradual and strong promotion of hypocotyl length in the *phyA* seedlings (from about 7.8 mm at R:FR = 0.148 to more than 10 mm at the lowest R:FR tested of 0.035) (Figures 2b, S2a). These results agree with the reported negative role of phyA in shade-induced hypocotyl elongation [10], that was most apparent at very low R:FR. W+FR light had a contrasting effect on hypocotyl elongation of *phyB* seedlings: at the highest R:FR (0.320) it did not increase the already W-grown long hypocotyls (about 8 mm). At lower R:FR (0.148 and 0.035), W+FR inhibited (rather than promoted) hypocotyl elongation compared to W-grown seedlings. As a result, at very low R:FR (i.e., the lowest R:FR used of 0.035), W+FR-grown *phyB* hypocotyls were even shorter than those of Col-0 growing under W (Figures 2b, S2a). A mild inhibition of the *phyB* hypocotyl elongation by W+FR has also been observed previously, an effect attributed to the phyA-imposed inhibition of hypocotyl elongation in W+FR, quite apparent in the absence of phyB [17], [44], [45].

We next used an intensity of W of 30–35 µmol·m^−2^·s^−1^ in the PAR region (from now on, we will refer to this light as of “medium PAR”). The R:FR applied ranged from 0.324 (intermediate R:FR) to 0.030 (very low R:FR) (Figure 2c). W+FR light strongly induced hypocotyl elongation of Col-0 compared to the W-grown seedlings (about 4 mm) for R:FR between 0.324-0.091. Further reductions in the R:FR (i.e., low and very low R:FR) were less effective in promoting hypocotyl elongation (about 3 mm for R:FR between 0.067-0.030) (Figures 2c, S2b). W+FR light also strongly induced hypocotyl length of *phyA* seedlings; the progressive reduction of the R:FR resulted in a very strong promotion of hypocotyl length (from about 4.5 mm at R:FR = 0.324 to more than 7 mm at the lowest R:FR tested of 0.030). For *phyB* seedlings, W+FR light did not affect hypocotyl length at the highest R:FR used (0.324) compared to W-grown seedlings, but it was progressively more effective in inhibiting hypocotyl elongation at lower R:FR (from 0.143 to 0.038) (Figures 2c, S2b).

Finally, we used an intensity of W of 50–60 µmol·m^−2^·s^−1^ in the PAR region (we will refer to this light as of “high PAR”). The applied R:FR ranged from 0.463 to 0.041 (Figures 2d). As for the low and medium PAR experiments, W+FR light induced hypocotyl elongation of Col-0 compared to the W-grown seedlings depending on the R:FR applied: the progressive reduction of the R:FR resulted in longer hypocotyls (from about 1.5 mm at a R:FR = 0.463 to almost 2.5 mm at a R:FR of 0.074). Further reductions in the R:FR were equally effective in promoting hypocotyl elongation (for R:FR<0.074) (Figures 2d, S2c). W+FR light affected hypocotyl length of *phyA* and *phyB* seedlings essentially as described for low and medium PAR cases: the reduction of the R:FR resulted in a progressive promotion of *phyA* hypocotyl length (from almost 2 mm at R:FR = 0.463 to more than 6 mm at the lowest R:FR tested of 0.041) and a progressive inhibition of *phyB* hypocotyls (it did not affect the already long hypocotyls at R:FR of 0.463 and 0.205, but it inhibited hypocotyl elongation at low and very low R:FR of 0.124 to 0.041) (Figures 2d, S2c).

We next analyzed the hypocotyl response to very low R:FR light (very low R:FR of 0.043, low PAR) of *phyA* and *phyB* mutant seedlings in the L*er* background. As shown in Figure S3, the hypocotyl elongation response of these L*er* genotypes was similar to the one observed in Col-0, confirming that similar effects were observed in other genetic backgrounds. Together, these results led us to conclude that (1) the reported negative role of phyA in the shade-induced hypocotyl elongation [10], [13] becomes more apparent at very low R:FR (0.05-0.03) and (2) it is qualitatively independent of the range of PAR intensity tested.

### The timing of treatment with very low R:FR strongly affects the hypocotyl elongation response

We also addressed the influence of the timing and duration of the W+FR treatment on the hypocotyl elongation response of Col-0, *phyA* and *phyB* seedlings. Seedlings were grown at low PAR and submitted to a low R:FR of 0.059, conditions that allow distinction between the hypocotyl phenotypes of all three genotypes (Figure 2b). When W+FR was applied from day 2 after germination (2W+5WF), hypocotyl length of the analyzed genotypes was affected as observed before: Col-0 hypocotyls were poorly responsive, and *phyA* and *phyB* hypocotyl elongation was strongly promoted and inhibited respectively. When W+FR was applied from days 3 or 4 from germination (3W+4WF and 4W+3WF), Col-0 and *phyB* hypocotyls elongated significantly more than those grown under 2W+5WF, the promotion of elongation being more obvious for *phyB* seedlings. By contrast, *phyA* hypocotyls elongated significantly less than those grown under 2W+5WF (Figure 3a). When W+FR was applied from day 7 after germination (7W+1WF), hypocotyl length of Col-0 and *phyA* seedlings was modestly promoted although *phyA* hypocotyls still were more responsive than those of Col-0 seedlings; by contrast *phyB* seedlings were unaffected (Figure 3b). These results indicated that shade-induced phyA repression of hypocotyl elongation was operative during the entire time of exposure to W+FR (from days 2 to 8). However, based on the effect of the timing of the W+FR application on the *phyA* and *phyB* final hypocotyl elongation, it seems that the repression was stronger at the early stages of seedling development, i.e., from days 2 to 4.

We also investigated whether the elongation in the *phyA* and *phyB* mutants occurred at the same time along the course of seedling development. Wild-type and *phyA* seedlings were grown at a PAR of 20–25 µmol·m^−2^·s^−1^ (medium PAR) and exposed to W+FR of R:FR of 0.083 (low R:FR) from day 2 to day 7 after germination, and *phyB* seedlings were grown from germination under W of the same PAR. Under these conditions we expected a noticeable hypocotyl elongation of all three genotypes. As shown in Figure 4a, hypocotyl length was recorded in 3-, 5- and 7-day-old seedlings. *phyA* hypocotyls were already longer than those of *phyB* and Col-0 on day 3 (in our growth conditions, 2-day-old hypocotyls of all three genotypes are still emerging from the seeds and, therefore, their length is close to 0 mm). Although elongation in both *phyA* and *phyB* seedlings was sustained along the whole period, *phyA* hypocotyls elongated more from days 3 to 5 (d3–d5, 3.01 mm; d5–d7, 2.35 mm), whereas *phyB* hypocotyls elongated substantially more from days 5 to 7 (d3–d5, 1.65 mm; d5–d7, 2.75 mm). In addition, Col-0 hypocotyls elongated more from days 5 to 7 (d3–d5, 0.63 mm; d5–d7, 1.36 mm), suggesting that under the W+FR conditions applied its elongation was strongly inhibited from days 2 to 5, the same time window in which *phyA* hypocotyls elongated more. These results are consistent with our previous conclusion that phyA-mediated repression was stronger at the early stages of seedling development.

### Treatment with very low R:FR stabilizes phyA levels

phyA is abundant in etiolated tissues but is light-labile and so is rapidly depleted in light-treated tissues. The long hypocotyl phenotype of *phyA* seedlings grown under W+FR of very low R:FR suggested that phyA may be more abundant under very low R:FR in Col-0 than under high R:FR light. To test this possibility, phyA levels were analyzed by western blot in 5-day-old etiolated Col-0 seedlings exposed to W (PAR of 20–25 µmol·m^−2^·s^−1^, R:FR = 2.5) and W+FR (same PAR, very low R:FR of 0.038) for 4 h (Figure 5a). As shown in Figure 5b, phyA levels declined after exposure to either W or W+FR light. However, in extracts from W+FR-exposed seedlings, phyA levels were higher (Figure 5c). Longer exposure times of W+FR (8 h) showed a similar tendency (Figure S4). Altogether, our data indicate that the balance between phyA synthesis and degradation in seedlings is affected by the R:FR of the incoming light, whereby light of very low R:FR has a milder destabilizing effect compared to W light of high R:FR.

## Discussion

As mentioned above, nearby vegetation selectively reflects FR, thus lowering the R:FR, a signal that induces plant responses in anticipation of neighboring vegetation becoming a competitive threat [46]. If, despite these responses, neighboring vegetation directly shades the plant, light quantity becomes limiting, i.e., there is a reduction in the amount of radiation active in photosynthesis (i.e., PAR, between 400–700 nm), resulting in additional or more dramatic SAS responses [27]. Measurements by different authors of different natural light environments agree with this view [1]–[4], [6], [47], [48]. In the laboratory, conditions that mimic plant proximity before actual canopy shading occurs (i.e., with lowered R:FR only, without changing PAR) have been termed *simulated shade*, and those that mimic natural situations when canopy closure occurs (which reduce both R:FR and PAR), have been termed *canopy shade* [25], [28]. We have shown here that perception of these types of light-environment are genetically distinguishable by analyzing the hypocotyl elongation of *phyA* and *phyB* mutant seedlings in response to light of different R:FR, even without altering the PAR intensity: the inhibitory effect of phyA is readily observed under very low R:FR by (1) the conspicuous hypocotyl elongation of the *phyA* seedlings compared to the wild-type, and (2) the strong inhibition of the long-hypocotyl phenotype of the *phyB* seedlings. However, our data reveal a dichotomy in responsiveness across the range of R:FR tested. As mentioned, these phenotypes are observed under the lowest R:FR ratios (e.g. about 0.05-0.03) in our W+FR experiments here (Figure 2). However, these responses are essentially absent under our intermediate R:FR treatments (about 0.5-0.3), whereby *phyA* hypocotyls behave almost as wild-type ones and the long *phyB* hypocotyls are unaffected by the simulated shade signal (Figures 2, S2, 6a).

**Figure 6:**
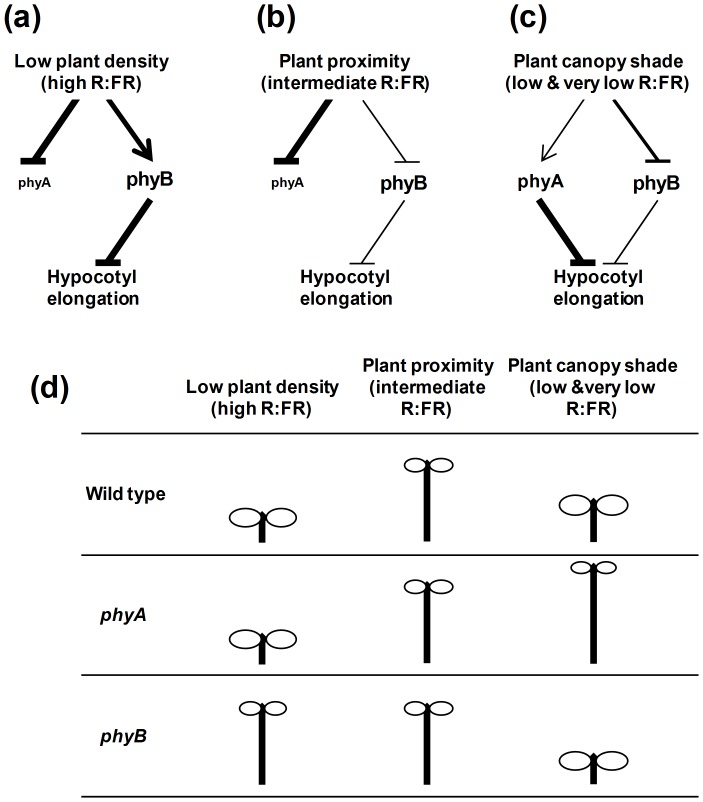
Model depicting the antagonistic effect of phyA and phyB on the shade-induced hypocotyl elongation. (**a**) Under low plant density, the high R:FR induces phyA degradation and stimulates the phyB active Pfr form, which strongly inhibits hypocotyl elongation. (**b**) In close proximity of vegetation, phyA degradation still occurs, but the low R:FR displaces the photoequilibrium of phyB towards the inactive Pr form, causing hypocotyls to elongate. (**c**) Under a plant canopy, the low or very low R:FR still displaces the photoequilibrium of phyB towards the inactive Pr form that stimulates hypocotyls to elongate. However, under these conditions phyA is stabilized, particularly at the beginning of the seedling emergence; as a consequence, phyA signaling is enhanced, thereby counteracting the inhibitory effect of the absence of active phyB, so that hypocotyls elongate only moderately. (**d**) Summary of phenotypes shown by the wild-type, *phyA* and *phyB* seedlings growing under the light conditions indicated in **a**, **b** and **c**.

The antagonistic role of phyA in the SAS regulation has been noted previously when examining seedling deetiolation using natural shade conditions (provided by densely-grown plants of common wheat), and it was shown to be important for seedling establishment and survival under these specific conditions: *phyA* deficient mutants displayed extreme elongation growth, poor cotyledon development (phenotypes similar to those observed here under very low R:FR, i.e. R:FR = 0.05-0.03) (Figures 2, S3) and a lower survival rate than wild-type seedlings [11]. Our data show that the inhibitory role of phyA occurs independently of the range of PAR levels tested here (Figure 2), suggesting that the level of PAR in the incident light, although broadly recognized to modulate plant development (Figure 1), does not alone contain the essential differential information between these two extreme types of R:FR conditions (i.e. simulated and canopy shade). Hence, the R:FR signal alone seems sufficient to differentiate between plant proximity (mild reductions in the R:FR, without PAR decrease, defined here as “intermediate R:FR”) and dense, direct canopy shade (strong reductions in the R:FR signal, defined here as “very low R:FR”). In this regard, *phyA* hypocotyls progressively elongated from intermediate to very low R:FR, and at the lowest levels tested they were much longer than those of *phyB* seedlings under W (high R:FR) (Figures 2, S2, S3), providing additional evidence for the effectiveness of the information contained in the R:FR signal. In addition, it suggests that the strong promotion of hypocotyl elongation observed in the *phyA* mutant background is due to the inactivation of the Pfr form of phyB and other photostable phys induced by the very low R:FR light treatment. It might be argued that the PAR intensities employed in these experiments are low compared to what might be observed in nature (e.g., 750 µmol·m^−2^·s^−1^ in a clear midday in Buenos Aires, Argentina) [11]. Nonetheless, under natural dense canopies, PAR intensity might be reduced to less than 0.5% at the solar zenith, reaching even lower intensities than the ones employed in this work (1 µmol·m^−2^·s^−1^). Under these low PAR intensities, the R:FR results in values similar to those described here as low (0.10±0.02; Table S1) [11], [49], [50]. Therefore, lower R:FR values might be reached at other times of the day or latitudes. It is interesting to note that in chlorophyll rich organs, such as leaves, light absorption from the ultraviolet to the visible region by chlorophyll a leads to the emission (by fluorescence) of FR light [51]. Therefore, leaf chlorophylls can actively contribute to create low and very low R:FR signals in natural deep-shaded environments.

During the first week of seedling emergence and development, the inhibitory role of phyA is very apparent and easily observed at the beginning of this period (from days 2 to 5) when the potential to elongate is very high. Once this potential is diminished, the role of phyA also becomes less relevant (Figures 3, 4) [13]. Seedlings grown under photoperiodic conditions also respond to transient (2 h) low R:FR, which has revealed that there is also a circadian component in phyA action: whereas simulated shade given at subjective dusk increases hypocotyl length, when given at subjective dawn leads to a small inhibition of hypocotyl elongation (compared to untreated seedlings), an antagonistic effect of low R:FR shown to be dependent on phyA [18]. PhyA also (1) inhibits hypocotyl elongation under short-day conditions [10], under continuous R [52], [53], or under continuous W of high R:FR but low PAR (Figure 1) and (2) promotes cotyledon expansion [52], [54], likely also at the early stages of seedling development. However, the role of phyA in light-grown plants is not restricted to these early stages of the seedling development. In adult plants grown under short-day conditions, phyA has also a role in suppressing internode growth and leaf elongation [54].

The various roles of phyA in light-grown seedlings and plants are consistent with the evidence that, although phyA levels have been strongly decreased in these light-exposed tissues, they are not reduced to zero [3], [55]. During seedling de-etiolation, phyA activation by FR results from the fact that continuous monochromatic FR light establishes and maintains a small fraction of the phyA population in the Pfr form, operating via the FR-HIR, over an extended period [3], [23], [56]. Indeed, although phyA is photolabile, FR-grown seedlings retain phyA at higher levels than R-grown seedlings as determined by western analyses blot analyses [55], [57]. Also, under light/dark cycles, phyA accumulates during the night and is rapidly degraded during the day [55], which might explain the long hypocotyl phenotype of *phyA* seedlings grown under short-days conditions [10] and the absence of growth inhibition in *phyA* seedlings when a transient low R:FR treatment is given at subjective dawn [18]. We have observed that phyA is degraded under W in a R:FR-dependent manner: under very low R:FR conditions phyA is more stable than under high R:FR (Figures 5, S4). The rapid increase in the expression of *PHYA* in response to low R:FR light very likely contributes to the observed maintenance of high phyA protein levels [44]. Therefore, it seems likely that in fully de-etiolated seedlings the activation of phyA by W+FR of very low R:FR maintains a small fraction of phyA cycling in the active Pfr form that likely results in the observed suppression of the hypocotyl elongation [13], [54], [58].

It has been discussed in the literature that certain aspects of seedling SAS and de-etiolation affect the same traits but in opposite directions, such as accelerated hypocotyl cell elongation, retarded cotyledon expansion and reduced photosynthetic pigment accumulation. Indeed, gene-expression analyses provided initial molecular evidence for this view [17] and the continuous modulation by the phy-PIF signaling system has been implicated [21]. The analyses of seedling de-etiolation using Arabidopsis *phyA* and *phyB* mutants have established that both phytochromes have roles in seedling de-etiolation: phyA is the only photoreceptor responsible for the response of seedlings to continuous monochromatic FR, whereas phyB is mainly responsible for the responses of seedlings to continuous monochromatic R (Figure S5). The complementary actions of phyA and phyB in this process has been considered to provide optimum regulation of seedling growth after emergence from the soil [23]. We show here that during the SAS response of seedlings, phyB is deactivated by shade of intermediate, low and very low R:FR, whereas phyA is only strongly activated by shade of low, and very low R:FR, partly because of its higher levels. As a result, the phyA-Pfr produced and sustained in a cycling state strongly inhibits hypocotyl elongation via the FR-HIR activity of this phy [13], [56] (Figure 6d). The differential effects of the *phyA* and *phyB* mutants on this process genetically defines the operation of two different pathways in SAS regulation [13], an additional similarity between the SAS and de-etiolation responses (Figures 6, S5). In the natural environment, continuous monitoring of the R:FR will determine the participation of phyA in the response to shade; when the R:FR is very low (such as in deep shade), phyA activation will prevent seedlings from exhibiting excessive elongation mediated principally by deactivation of phyB [58]. Whereas the overlapping actions of phyA and phyB will substantially promote de-etiolation in sparse vegetation [12], the antagonistic action of phyA and phyB will ensure the optimum elongation under deep shade, conditions in which R:FR can be strongly reduced partly due to the active emission of FR by the chlorophyll from the leaves [51].

Collectively, our data provide evidence that phyA functions in natural light environments to attenuate the SAS in response to direct canopy shading, but not to simple neighbor-proximity. This deduction refines the existing concept that phyA can “antagonize” the SAS via the FR-HIR [2], [10], [13], and supports the notion that plants have evolved a sophisticated intrafamily photosensory attenuation mechanism that can discriminate between the threat and imposition of competition for PAR by neighboring vegetation. This dual-track mechanism provides young seedlings with the capacity for both rapid elongation upon sensing of impending competition (intermediate R:FR) (the “neighbor-detection response mode”), or attenuation of potentially deleterious excessive elongation upon direct interception of canopy shade (low or very low R:FR) (the “direct-shade response mode”).

## Supporting Information

Figure S1 **The** ***phyA-501*** **line is deficient in phyA.** (**a**) Scheme of the *PHYA* genomic structure and the site of insertion of the T-DNA in the SALK_014575 line; arrows indicate the approximate location of primers used for genotyping (LBb1, MSO31 and MSO32). (**b**) Steady-state levels of phyA measured by protein blot. Immunoblot detection of phyA (upper panel) and tubulin (TUB, lower panel) levels in extracts from Col-0 and *phyA-501* seedlings grown in darkness for 5 days. Bands were detected as indicated in Figure 5b. (**c**) Hypocotyl length in 4-day-old Col-0 and *phyA-501* seedlings grown in darkness, continuous FR (3.7 µmol·m^−2^·s^−1^) and R (12.8 µmol·m^−2^·s^−1^), as shown in the upper part of the panel. Mean and SE values represent at least 25 seedlings from each treatment. Asterisks indicate significant differences (*P<0.05; **P<0.01) relative to control seedlings (Col-0) grown under the same conditions.
(PDF)

Figure S2 **Effect of different R:FR on hypocotyl elongation of wild-type,** ***phyA-501*** **and** ***phyB-9*** **seedlings under low (a), medium (b) or high (c) PAR represented as the difference (left panels) or the ratio (right panels) between values under W+FR and W.** Data were recalculated from the experiments generated for Figure 2. Values are the mean and SD from the 3 independent experiments.
(PDF)

Figure S3 **Effect of very low R:FR ratios on hypocotyl elongation of wild-type (L** ***er*** **),** ***phyA*** **and** ***phyB*** **seedlings under high light intensity.** Seedlings were germinated and grown (PAR was of 15–16 µmol·m^−2^·s^−1^) as indicated in Figure 2a. Under W+FR, R:FR was 0.043. Mean and SE values represent at least 25 seedlings from each light treatment. Asterisks indicate significant differences (*P<0.05; **P<0.01) relative to control seedlings (L*er*) grown under the same light conditions.
(PDF)

Figure S4 **Levels of phyA are stabilized by very low R:FR treatments.** (**a**) phyA levels were detected in extracts from Col-0 seedlings grown in darkness for 5 days and then either transferred to W or W+FR for 4 and 8 hours. Symbols indicate the harvest time of the plant material. (**b**) Immunoblot detection of steady-state levels of phyA (upper panel) and TUB (lower panel) in extracts from seedlings grown as indicated in (**a**). Bands were detected as indicated in Figure 5b.
(PDF)

Figure S5 **Schematic summary of the phenotypes of wild-type,** ***phyA*** **and** ***phyB*** **seedlings grown in the dark or monochromatic R o FR light (indicated at the top).**
(PDF)

Table S1 **Terminology of the various R:FR regimes applied in this work and its proposed equivalence under natural conditions.**
(PDF)
