# Supplementary material for: The Shade Avoidance Syndrome in Arabidopsis: The Antagonistic Role of Phytochrome A and B Differentiates Vegetation Proximity and Canopy Shade
Source: PLoS One. 2014 Oct 21;9(10):e109275. doi: 10.1371/journal.pone.0109275 (PMC4204825; doi:10.1371/journal.pone.0109275)
Supplement: Figure S1 — The phyA-501 line is deficient in phyA. (a) Scheme of the PHYA genomic structure and the site of insertion of the T-DNA in the SALK_014575 line; arrows indicate the approximate location of primers used for genotyping (LBb1, MSO31 and MSO32). (b) Steady-state levels of phyA measured by protein blot. Immunoblot detection of phyA (upper panel) and tubulin (TUB, lower panel) levels in extracts from Col-0 and phyA-501 seedlings grown in darkness for 5 days. Bands were detected as indicated in Figure 5b. (c) Hypocotyl length in 4-day-old Col-0 and phyA-501 seedlings grown in darkness, continuous FR (3.7 µmol·m−2·s−1) and R (12.8 µmol·m−2·s−1), as shown in the upper part of the panel. Mean and SE values represent at least 25 seedlings from each treatment. Asterisks indicate significant differences (*P<0.05; **P<0.01) relative to control seedlings (Col-0) grown under the same conditions. (PDF) [file pone.0109275.s001.pdf]

Figure S1

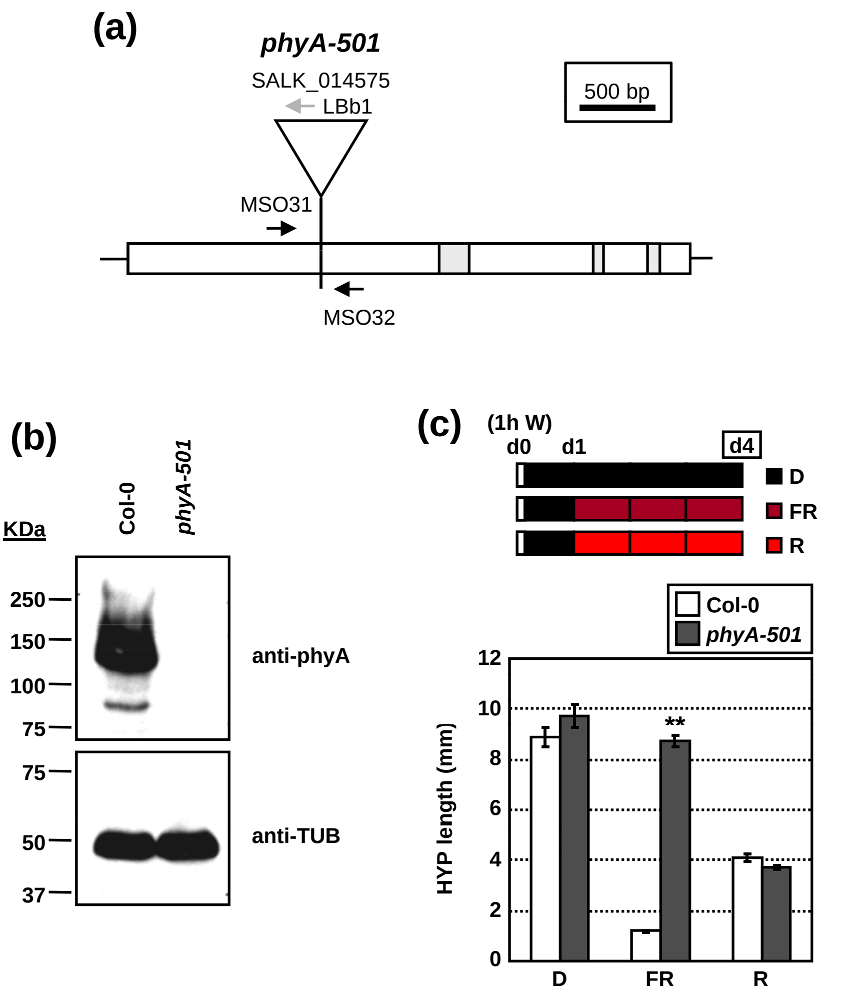

Figure S1. The *phyA-501* line is deficient in phyA. (**a**) Scheme of the *PHYA* genomic structure and the site of insertion of the T-DNA in the SALK_014575 line; arrows indicate the approximate location of primers used for genotyping (LBb1, MSO31 and MSO32). (**b**) Steady-state levels of phyA measured by protein blot. Immunoblot detection of phyA (upper panel) and tubulin (TUB, lower panel) levels in extracts from Col-0 and *phyA-501* seedlings grown in darkness for 5 days. Bands were detected as indicated in Fig. 5b. (**c**) Hypocotyl length in 4-day-old Col-0 and *phyA-501* seedlings grown in darkness, continuous FR (3.7 $\mu mol \cdot m^{-2} \cdot s^{-1}$) and R (12.8 $\mu mol \cdot m^{-2} \cdot s^{-1}$), as shown in the upper part of the panel. Mean and SE values represent at least 25 seedlings from each treatment. Asterisks indicate significant differences (*P<0.05; **P<0.01) relative to control seedlings (Col-0) grown under the same conditions.
