# Supplementary material for: The Shade Avoidance Syndrome in Arabidopsis: The Antagonistic Role of Phytochrome A and B Differentiates Vegetation Proximity and Canopy Shade
Source: PLoS One. 2014 Oct 21;9(10):e109275. doi: 10.1371/journal.pone.0109275 (PMC4204825; doi:10.1371/journal.pone.0109275)
Supplement: Figure S2 — Effect of different R:FR on hypocotyl elongation of wild-type, phyA-501 and phyB-9 seedlings under low (a), medium (b) or high (c) PAR represented as the difference (left panels) or the ratio (right panels) between values under W+FR and W. Data were recalculated from the experiments generated for Figure 2. Values are the mean and SD from the 3 independent experiments. (PDF) [file pone.0109275.s002.pdf]

Figure S2

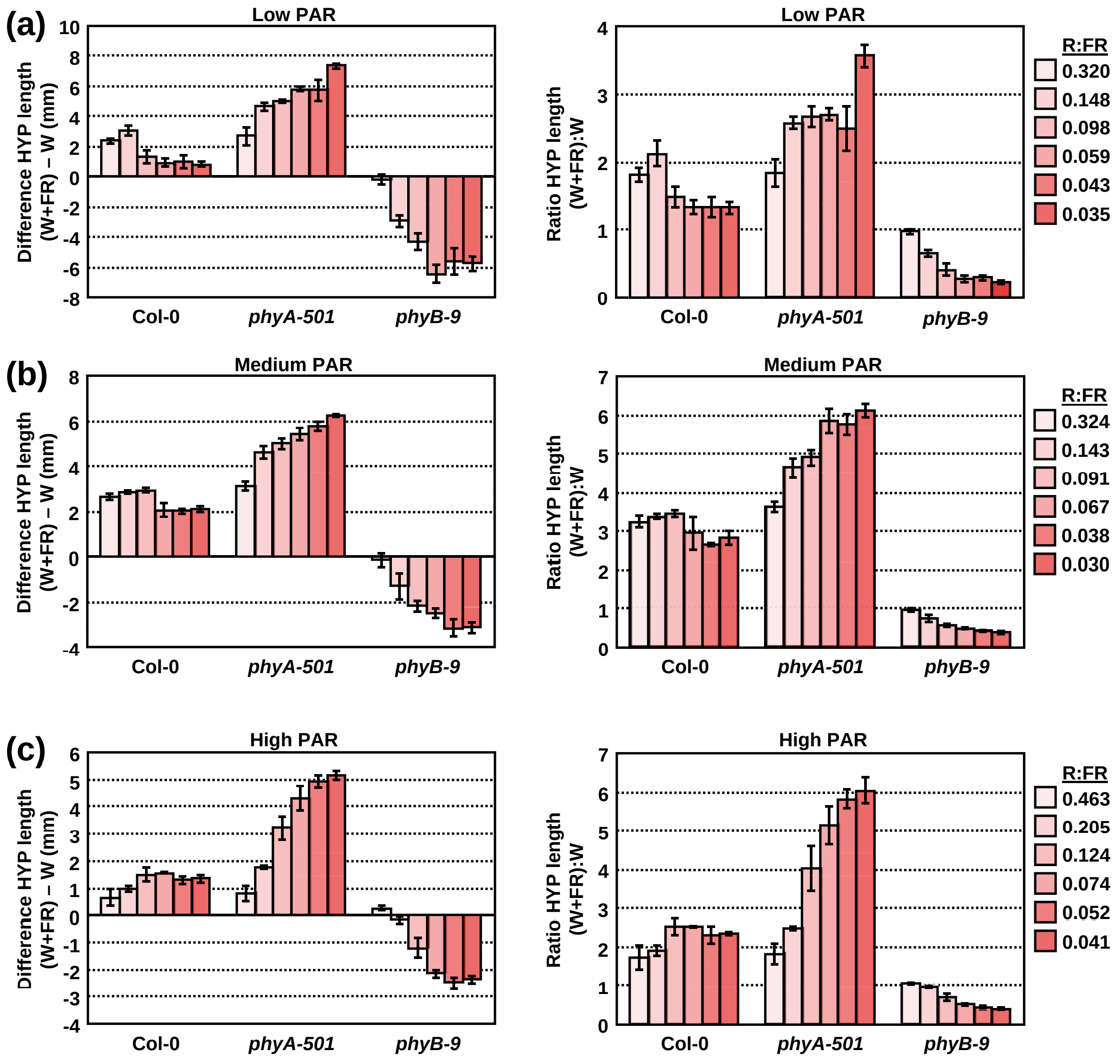

Figure S2. Effect of different R:FR on hypocotyl elongation of wild-type, *phyA-501* and *phyB-9* seedlings under low **(a)**, medium **(b)** or high **(c)** PAR represented as the difference (left panels) or the ratio (right panels) between values under W+FR and W. Data were recalculated from the experiments generated for Figure 2. Values are the mean and SD from the 3 independent experiments.
