# Supplementary material for: The Shade Avoidance Syndrome in Arabidopsis: The Antagonistic Role of Phytochrome A and B Differentiates Vegetation Proximity and Canopy Shade
Source: PLoS One. 2014 Oct 21;9(10):e109275. doi: 10.1371/journal.pone.0109275 (PMC4204825; doi:10.1371/journal.pone.0109275)
Supplement: Figure S3 — Effect of very low R:FR ratios on hypocotyl elongation of wild-type (L er ), phyA and phyB seedlings under high light intensity. Seedlings were germinated and grown (PAR was of 15–16 µmol·m−2·s−1) as indicated in Figure 2a. Under W+FR, R:FR was 0.043. Mean and SE values represent at least 25 seedlings from each light treatment. Asterisks indicate significant differences (*P<0.05; **P<0.01) relative to control seedlings (Ler) grown under the same light conditions. (PDF) [file pone.0109275.s003.pdf]

Figure S3

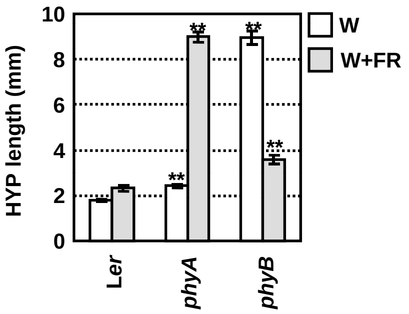

Figure S3. Effect of very low R:FR ratios on hypocotyl elongation of wild-type (L*er*), *phyA* and *phyB* seedlings under high light intensity. Seedlings were germinated and grown (PAR was of 15-16 µmol·m^{-2}·s^{-1}) as indicated in Fig. 2a. Under W+FR, R:FR was 0.043. Mean and SE values represent at least 25 seedlings from each light treatment. Asterisks indicate significant differences (*P<0.05; **P<0.01) relative to control seedlings (L*er)* grown under the same light conditions.
