# Supplementary material for: The Shade Avoidance Syndrome in Arabidopsis: The Antagonistic Role of Phytochrome A and B Differentiates Vegetation Proximity and Canopy Shade
Source: PLoS One. 2014 Oct 21;9(10):e109275. doi: 10.1371/journal.pone.0109275 (PMC4204825; doi:10.1371/journal.pone.0109275)
Supplement: Figure S4 — Levels of phyA are stabilized by very low R:FR treatments. (a) phyA levels were detected in extracts from Col-0 seedlings grown in darkness for 5 days and then either transferred to W or W+FR for 4 and 8 hours. Symbols indicate the harvest time of the plant material. (b) Immunoblot detection of steady-state levels of phyA (upper panel) and TUB (lower panel) in extracts from seedlings grown as indicated in (a). Bands were detected as indicated in Figure 5b. (PDF) [file pone.0109275.s004.pdf]

Figure S4

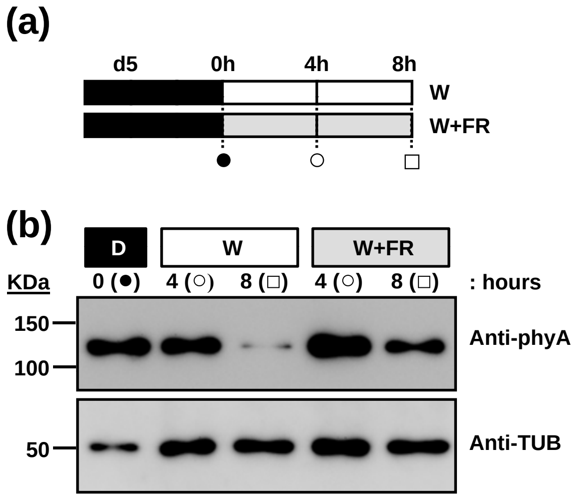

Figure S4. Levels of phyA are stabilized by very low R:FR treatments. (**a**) phyA levels were detected in extracts from Col-0 seedlings grown in darkness for 5 days and then either transferred to W or W+FR for 4 and 8 hours. Symbols indicate the harvest time of the plant material. (**b**) Immunoblot detection of steady-state levels of phyA (upper panel) and TUB (lower panel) in extracts from seedlings grown as indicated in (**a**). Bands were detected as indicated in Fig. 5b.
