# Supplementary material for: The Shade Avoidance Syndrome in Arabidopsis: The Antagonistic Role of Phytochrome A and B Differentiates Vegetation Proximity and Canopy Shade
Source: PLoS One. 2014 Oct 21;9(10):e109275. doi: 10.1371/journal.pone.0109275 (PMC4204825; doi:10.1371/journal.pone.0109275)
Supplement: Figure S5 — Schematic summary of the phenotypes of wild-type, phyA and phyB seedlings grown in the dark or monochromatic R o FR light (indicated at the top). (PDF) [file pone.0109275.s005.pdf]

Figure S5

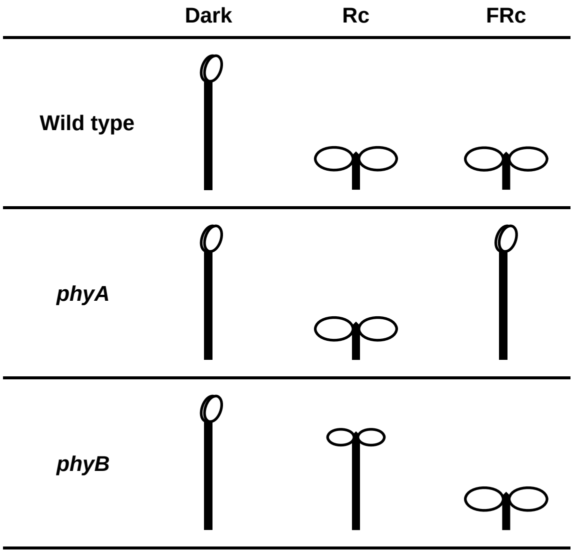

Figure S5. Schematic summary of the phenotypes of wild-type, *phyA* and *phyB* seedlings grown in the dark or monochromatic R o FR light (indicated at the top).
