# Supplementary material for: The Shade Avoidance Syndrome in Arabidopsis: The Antagonistic Role of Phytochrome A and B Differentiates Vegetation Proximity and Canopy Shade
Source: PLoS One. 2014 Oct 21;9(10):e109275. doi: 10.1371/journal.pone.0109275 (PMC4204825; doi:10.1371/journal.pone.0109275)
Supplement: Table S1 — Terminology of the various R:FR regimes applied in this work and its proposed equivalence under natural conditions. (PDF) [file pone.0109275.s006.pdf]

Table S1. Terminology of the various R:FR regimes applied in this work and its proposed equivalence under natural conditions.

**Change in R:FR**	**R:FR nomenclature**	**R:FR value**	**Equivalent in nature to:**	**How it is generated in the lab**
No change	High R:FR	>1.5	Open sun	W
Moderate decrease	Intermediate R:FR	0.50-0.30	Proximal neighbors without direct shading	W+FR
Substantial decrease	Low R:FR	0.29-0.06	Moderate direct plant shade	W+FR
Very large decrease	Very low R:FR	0.059-0.030	Dense direct plant shade	W+FR
